# LncRNA:DNA triplex-forming sites are positioned at specific areas of genome organization and are predictors for Topologically Associated Domains

**DOI:** 10.1186/s12864-021-07727-7

**Published:** 2021-05-28

**Authors:** Benjamin Soibam, Ayzhamal Zhamangaraeva

**Affiliations:** Computer Science and Engineering Technology, University of Houston-Downtown, One Main St, TX 77002 Houston, USA

**Keywords:** Long noncoding RNAs, TADs, Triplex structures, TAD-lncRNAs, RNA:DNA triplex, CTCF

## Abstract

**Background:**

Chromosomes are organized into units called topologically associated domains (TADs). TADs dictate regulatory landscapes and other DNA-dependent processes. Even though various factors that contribute to the specification of TADs have been proposed, the mechanism is not fully understood. Understanding the process for specification and maintenance of these units is essential in dissecting cellular processes and disease mechanisms.

**Results:**

In this study, we report a genome-wide study that considers the idea of long noncoding RNAs (lncRNAs) mediating chromatin organization using lncRNA:DNA triplex-forming sites (TFSs). By analyzing the TFSs of expressed lncRNAs in multiple cell lines, we find that they are enriched in TADs, their boundaries, and loop anchors. However, they are evenly distributed across different regions of a TAD showing no preference for any specific portions within TADs. No relationship is observed between the locations of these TFSs and CTCF binding sites. However, TFSs are located not just in promoter regions but also in intronic, intergenic, and 3’UTR regions. We also show these triplex-forming sites can be used as predictors in machine learning models to discriminate TADs from other genomic regions. Finally, we compile a list of important “TAD-lncRNAs” which are top predictors for TADs identification.

**Conclusions:**

Our observations advocate the idea that lncRNA:DNA TFSs are positioned at specific areas of the genome organization and are important predictors for TADs. LncRNA:DNA triplex formation most likely is a general mechanism of action exhibited by some lncRNAs, not just for direct gene regulation but also to mediate 3D chromatin organization.

**Supplementary Information:**

The online version contains supplementary material available at 10.1186/s12864-021-07727-7.

## Background

Chromatin conformation capture experiments such as Hi-C have shown that chromosomes are organized into units called topologically associated domains (TADs) which are separated by boundaries enriched in CCCTC-binding factor (CTCF) binding sites and highly transcribed genes [1, 2]. TADs are biologically significant because disruption of the boundaries affects the expression of nearby genes and can also be linked to diseases [3–6].

The mechanism for the specification or formation of TADs is not completely understood and is an active area of research. Some recent studies have suggested a linear tracking mechanism called the “loop extrusion model” [7–9], which suggests that the specification of TADs may be a result of an interplay between chromatin, cohesin SMC complex, and CTCF binding sites at boundaries of TADs. However, some boundaries are CTCF independent and are resistant to the loss of CTCF [1, 10, 11]. In recent years, other factors have also been uncovered that may have a role in the formation of TADs such as type II DNA topoisomerase [12], YY1, and Mediator (together with cohesin) [13, 14]. Some TAD boundaries, which are independent of CTCF, may simply act as transitions between active and repressed chromatin regions or host promoters of newly transcribed genes [1, 15]. Therefore, mammalian TADs seem not to be always the result of CTCF/cohesin loops and could sometimes rather be defined by chromatin state and other factors.

Long noncoding RNAs (lncRNAs) are RNAs longer than 200 nucleotides (nt) that do not code for proteins. There is well-documented evidence that a growing number of lncRNAs have important biological functions [16]. One of the mechanisms through which lncRNAs exhibit their functions is by forming lncRNA:DNA triplex structures. For example, lncRNAs as *HOTAIR* [16, 17], *MEG3* [18], and *Fendrr* [19, 20] form triplex helices with DNA at promoter regions to influence gene expression. In the context of 3D topological genome organization, there is some indication that the triplex-forming mechanism may be used by lncRNAs (such as *Firre*) to mediate chromosomal contacts [17]. In this paper, we consider the idea that some lncRNAs localize to specific locations of the genome by forming RNA:DNA triplex structures, which allow lncRNAs to exert their functions to preserve, mediate the overall organization of the genome and hence may lead to specification or maintenance of TADs.

DNA binding factors such as CTCF and the Cohesin complex are enriched in TAD boundaries and play a role in the specification of the boundaries and domain loops [1, 2, 8, 18]. The expansion of transposons in the genome may also indirectly mediate TAD specification by contributing to CTCF binding [18–20]. SINEs transposons are enriched in TAD boundaries while LINEs transposons are depleted in those locations [18]. These studies indicate that factors contributing to the mediation of chromatin organization have non-random enrichment in specific areas of the chromatin in relationship to the overall 3D genome organization. Therefore, to investigate any potential role of lncRNA:DNA triplex-forming sites in 3D chromatin organization, we first set out to perform a genome-wide analysis of locations of triplex-forming sites of lncRNAs. We employ statistical methods and machine learning tools to test for enrichment of these sites in TADs, their boundaries, and loop anchors. A non-random enrichment cannot directly imply a biological role of the triplex sites in TADs specification. However, it will provide a compelling reason for further experiments and analysis to decipher the potential biological roles of lncRNAs in mediating genome chromatin organization via RNA:DNA triplex sites.

## Results

### Expressed LncRNAs

**Fig. 1:**
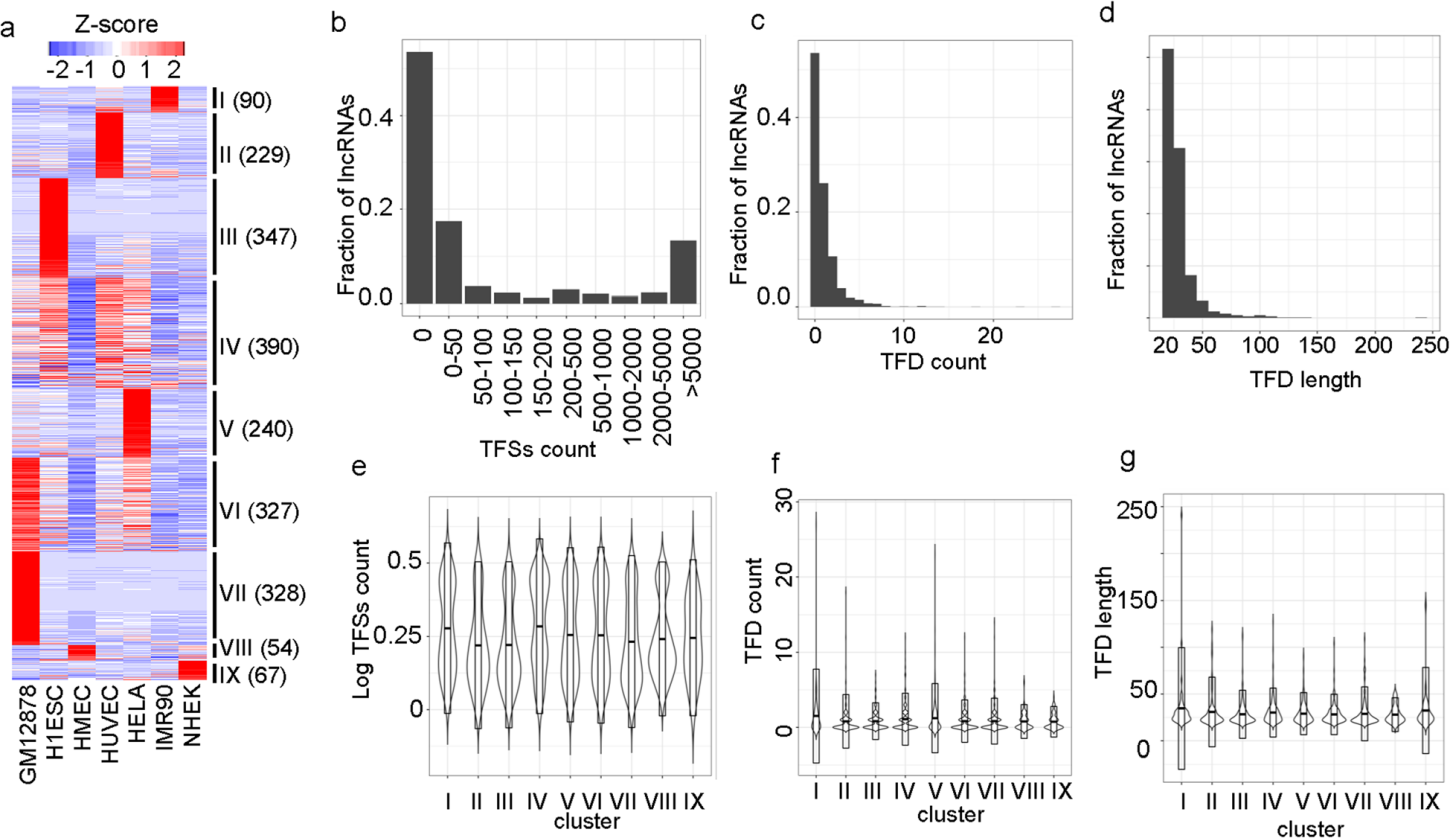
LncRNAs expression patterns and their triplex-forming sites. (**A**) Heatmap showing the clustering results of lncRNAs based on their expression across seven cell lines. Nine clusters are annotated next to the heatmap with Roman numerals. Gene count in each cluster is indicated in parentheses. The fraction of lncRNAs w.r.t triplex-forming sites (TFSs) count, triplex-forming domain (TFD) count, and triplex-forming domain length are shown in panels (**B**), (**C**), and (**D**), respectively. Violin plots of TFSs count, TFD count, and TFD length for lncRNAs belonging to different clusters identified in panel (**A**) are shown in panels (**E**), (**F**), and (**G)**, respectively

To investigate the triplex-forming sites of lncRNAs in a cell line of interest, we only considered the expressed lncRNAs in that cell line (Methods). This yielded 2,072 lncRNAs which were expressed in at least one of the seven human cell lines. We found 970, 853, 199, 773, 760, 322, and 325 lncRNAs which were expressed in cell lines GM12878, H1ESC, HMEC, HUVEC, HeLa, IMR90, and NHEK, respectively. To investigate the expression patterns of these 2,072 lncRNAs, their TPM values across the cell lines were clustered using Hierarchical Ordered Partitioning and Collapsing Hybrid (HOPACH) algorithm [21] (Fig. 1A). This revealed nine clusters of lncRNAs with distinct expression patterns (Fig. 1A). There were seven clusters, each one exhibited amplified expression in exactly one unique cell line (clusters I, II, III, V, VII, VIII, and IX for IMR90, HUVEC, H1ESC, HeLa, GM12878, HMEC, and NHEK, respectively) (Fig. 1A). There were only two clusters (clusters IV and VI) that showed nonspecific expression patterns (Fig. 1A). These observations resonate with previous reports of high cell and tissue specificity of lncRNAs [22, 23].

### Thousands of LncRNA:DNA triplex-forming sites

To determine the lncRNA:DNA triplex-forming sites (TFSs) of expressed lncRNAs for each cell line, we aligned the lncRNA sequences to the hg19 genome using triplexator tool [24] restricting the length of the triplex structures to a minimum length of 20 bp. Interestingly, about 54 % (1110 out of 2072) of lncRNAs did not form lncRNA:DNA TFSs (Fig. 1B). The remaining 962 lncRNAs which formed at least one lncRNA:DNA TFS fell into two main categories: the first group (17 % or 361 lncRNAs) had less than 50 TFSs, and the second group (13 % or 275) had more than 5000 TFSs (Fig. 1B). LncRNAs use short regions within their sequence to form the triplex structures with the double-stranded DNA. We call such regions Triplex forming domains (TFDs). The alignment results by triplexator tool contain information on the portions of lncRNAs that bind to the DNA. We found that even though lncRNAs have the potential to form many triplex sites throughout the genome, they had very few triplexes forming domains (TFDs) within their sequence (Fig. 1C). Out of the 962 lncRNAs which have TFDs, 541, 221, and 82 had 1, 2, and 3 TFDs, respectively (Fig. 1C). The majority of the TFDs have a length ranging between 20 nucleotides and 30 nucleotides (Fig. 1D). These results indicate that lncRNAs may harbor one or two specific short sequences (TFDs) that allow them to anchor to many sites in the DNA via a lncRNA: DNA triplex-forming mechanism.

Next, we checked the relationship between the triplex-forming potential of lncRNAs and clusters identified in Fig. 1A. We found no statistically significant dependence between the number of TFDs and TFSs of lncRNAs, length of the TFDs with their expression pattern identified in the 9 clusters (p-value > 0.08 using ANOVA test) (Fig. 1E and F, and G) suggesting a triplex-forming mechanism as a general mechanism followed by lncRNAs across multiple cell lines.

### Triplex-forming sites are enriched within topologically associated domains, their boundaries, and loop anchors more than expected, but they are evenly distributed across TADs

**Fig. 2:**
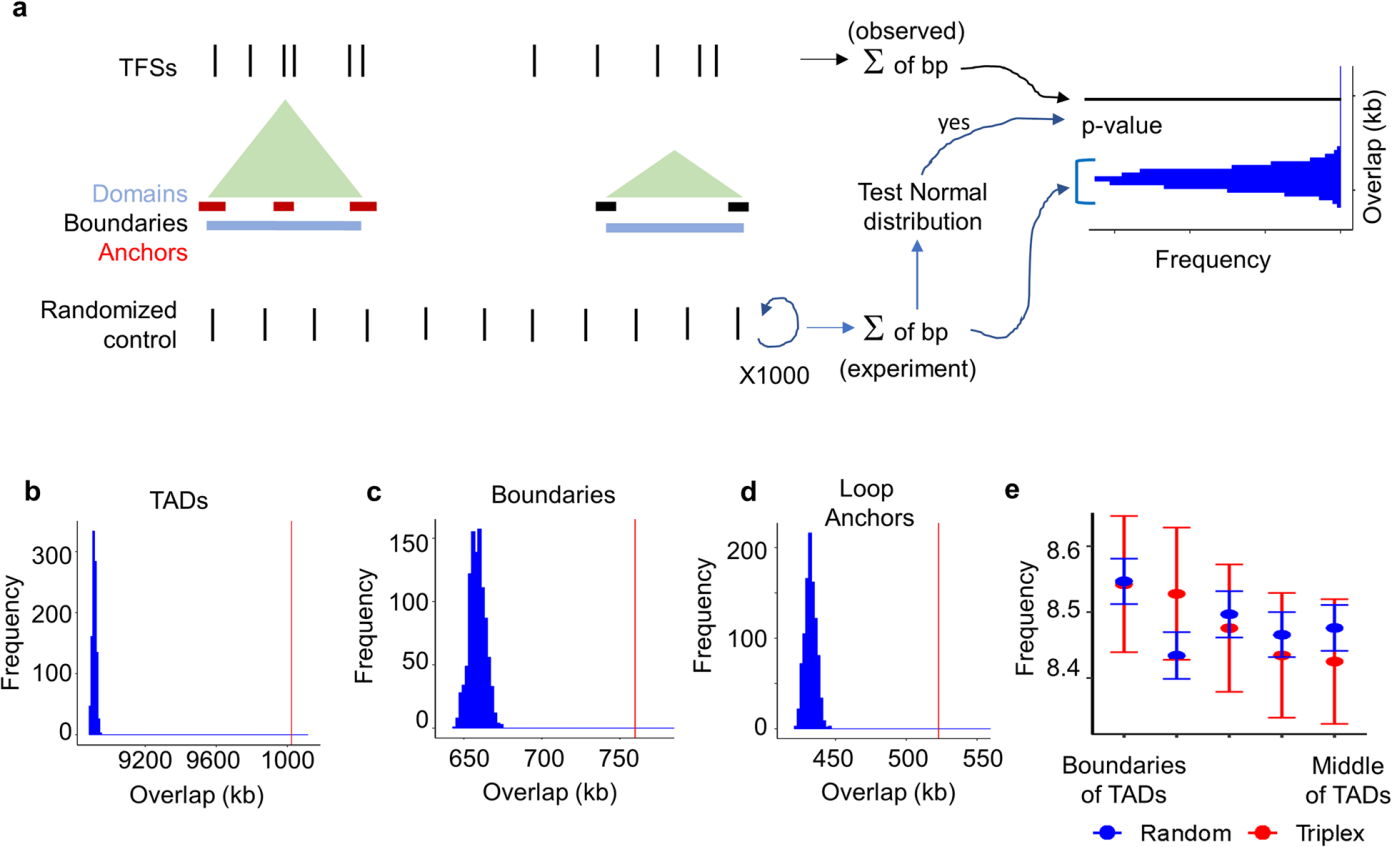
Triplex-forming sites (TFSs) are enriched in TADs, boundaries, and anchors but evenly distributed across TADs. (**A**) Illustration describing the procedure to perform a statistical test to check for the enrichment of TFSs in domains (or boundaries or loop anchors). The observed coverage of TFSs in all the domains (or boundaries or anchors) is the sum of all the base pairs in the domains (or boundaries or anchors) that overlap with the TFSs. Expected coverage is generated by randomly permuting the TFSs within the genome and computing the coverage of this random set with the domains (or boundaries or anchors). This random shuffling is performed 1000 times, for each shuffled set; an expected coverage is obtained to generate a distribution of expected coverage. These distributions are checked for normality using the Anderson-Darling normality test. Distribution of expected coverage (blue) versus the observed coverage (vertical red line) of TFSs in domains, boundaries, and anchors are shown in panels (**B**), (**C**), and (**D**), respectively for the HeLa cell line. (**E**) Frequencies of observed TFSs are evenly distributed across TADs and not significantly different from expected frequencies (*p*-value > 0.1 using Kolmogorov-Smirnov test). The graph is for the HeLa cell line

Next, we investigated the positions of TFSs relative to TADs to detect any positional preference. Genomic coordinates for TADs, their boundaries, and loop anchors were acquired from a previous study [1, 2] (Methods). In the seven cell lines, the TAD boundaries and loop anchors constitute a small fraction of the genome (between 1 and 6 %). In the majority of the cell lines close to 50 % of the genome is covered by TADs (Table S1 in Additional file 1). In IMR90 and H1ESC cell lines, about 65 and 83 % of the genome are covered by TADs, respectively (Table S1 in Additional file 1). To assess whether the lncRNA:DNA TFSs are enriched in TADs, we computed the observed coverage (or number of base pair overlaps) of TADs with the TFSs (Fig. 2A). Because of different coverages of the genome by TADs, we performed this separately for the cell lines. An expected coverage was generated by randomly positioning the TFSs within the genome and computing the coverage of this random set with the TADs Fig. 2A. This random shuffling was performed 1000 times, for each shuffled set; an expected coverage was obtained to generate a distribution of expected coverage. These distributions followed a normal distribution for all the seven cell lines (Anderson-Darling normality test: p-value > 0.01, Table S2 in Additional file 1). We found that in all the seven cell lines, the observed coverage of TFSs of lncRNAs with TADs was significantly higher than the expected coverage (p-value < 10^− 16^) (Fig. 2B and Fig. S1 in Additional file 1). Similarly, the observed coverage of TFSs with boundaries of TADs (Fig. 2C and Fig. S2 in Additional file 1) and loop anchors (Fig. 2D and Fig. S3 in Additional file 1) were significantly higher than the expected coverage in all the seven cell lines.

Next, we checked if there was a positional preference of the TFSs at specific locations across a TAD. This can inform if TSSs prefer regions close to the boundaries or away from them. For this, each TAD was divided into five bins of equal length. The frequencies of TFSs in the bins were computed. The TFSs were positioned randomly within the entire genome and frequencies of randomized regions in the five bins were also computed. We found that the TFSs were roughly evenly distributed across the entire length of a TAD (Fig. 2E, Fig. S4 in Additional file 1) and not significantly different from the random control (p-value > 0.1 using Kolmogorov-Smirnov test). This indicates no significant preference for TSSs for any specific region across a TAD.

### Triplex-forming sites occupancy correlates with the size of domains and is positioned distant from CTCF binding sites

**Fig. 3:**
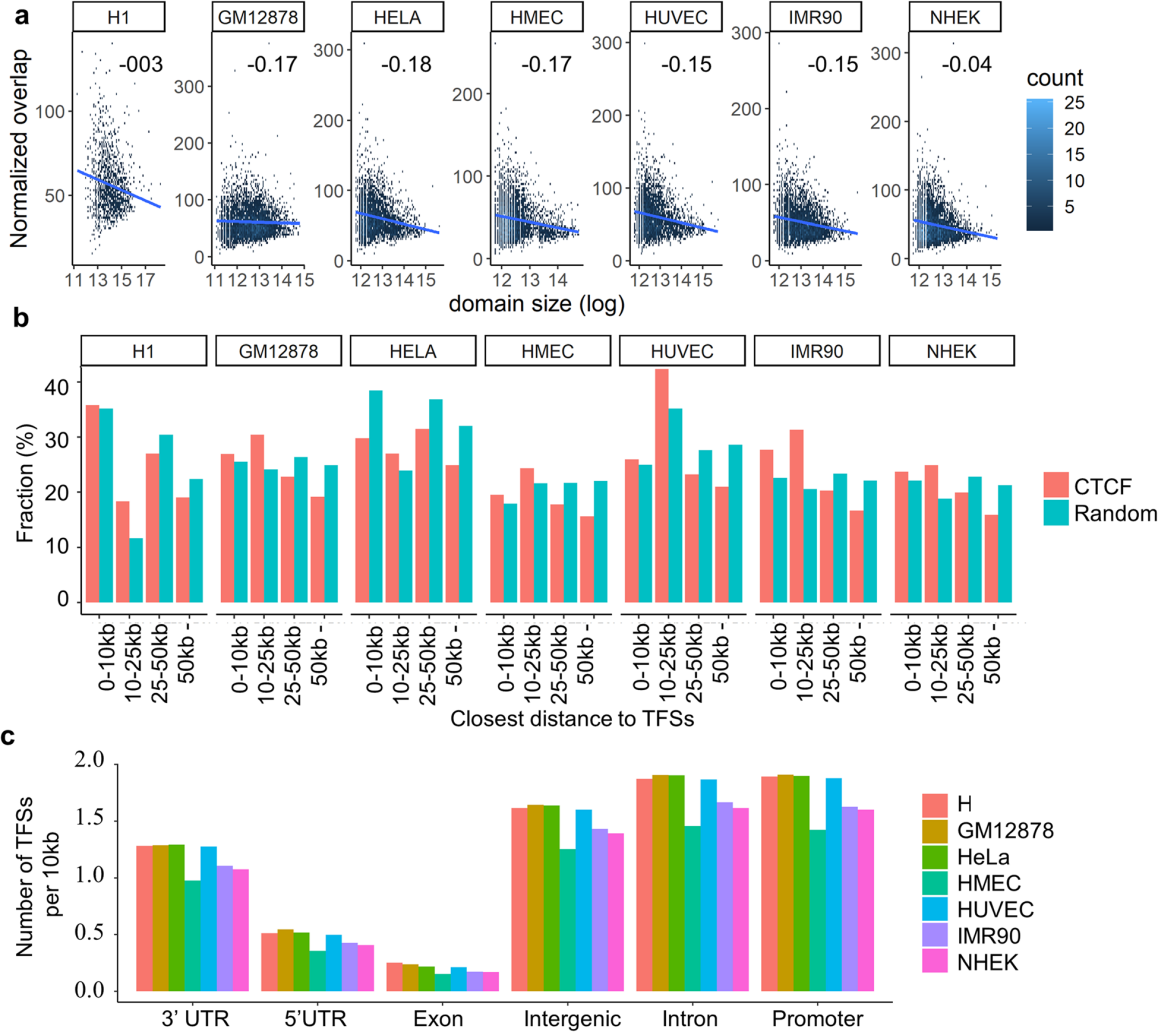
Relationships of Triplex forming sites with domain size, CTCF sites, and genomic annotation. (**A**) A small negative correlation between the size of domains (x-axis) and the normalized overlap between TFSs and TADs (y-axis). The Pearson correlation coefficients are indicated for each cell line. (**B**) Distances between closest pairs of CTCF sites and TFSs are not significantly different from random and TFSs (Chi-Square test, p-value > 0.1 for each cell line). The plots are histogram plots of the distances with four bins. (**C**) Genomic annotation (x-axis) of lncRNA:DNA TFSs reveal major fraction (y-axis) of them are in promoter, intronic, intergenic, and 3’UTR regions.

Next, we explore the relationship between the number of TFSs and the size of TADs. For this, we first normalized the coverage of TFSs in a TAD by the size of the TAD. Then the normalized coverages were compared to the corresponding sizes of TADs. There was a small negative linear correlation between the normalized coverage of TFSs and the size of TADs (Fig. 3A). When the same analysis was performed with the randomly positioned TFSs, no correlation between the normalized coverages and sizes of TADs was observed (Fig. S5 in Additional file 1). This suggests that TFSs are present in smaller domains at a moderate density compared to larger domains. CTCF is an insulator binding factor and has been linked to different properties of the 3D chromatin organization. To check the relationship between CTCF binding sites and TFSs, we computed a histogram plot of the distances between closest pairs of CTCF binding sites and TFSs using four bins (Fig. 3A). We also positioned the binding sites of CTCF randomly to obtain a set of randomized locations. The same histogram plot was constructed using the closest pairs of TFSs and randomized CTCF sites. We found no statistical difference between the two histogram plots (Chi-Square Test, p-value > 0.1 for all cell lines) (Fig. 3B) most likely because CTCF are preferred near boundaries while TFSs are roughly evenly distributed across TADs. Next, we investigated the densities of TFSs in different functional genomic elements (Methods). We found that the highest density of TFSs was in promoter or intronic regions with 2 TFSs for every 10 kb of a promoter or intronic region. Intergenic regions had a comparable (but slightly lower) density of 1.8 TFSs for every 10 kb interval. (Fig. 3C) compared to TFSs densities of 1.3, 0.5, and 0.2 TFSs for every 10 kb 3’UTR, 5’UTR, and exonic regions, respectively. The enrichment of TFSs in other functional genomic elements such as intergenic, intronic, and 3’UTR regions (not just in promoter regions) indicates a broader role of TFSs beyond direct gene regulation via protein-complex transportation to promoters.

### Triplex forming sites within TADs that are shared in many cell types are associated with early development processes

Next, we focused on the TFSs, which occur within domains present in all the 7 sets of human TADs. We required such TFSs to occur within a domain in each of the seven sets of human TADs. Pooling together the TFSs from all the cell lines that overlapped with at least one domain yielded 571,832 unique sites. Out of this, 17,589, 55,851, 7650, 1150, 8551, 7369, 4055 sites were specific to domains belonging to GM12878, H1, HeLa, HMEC, HUVEC, IMR90, and NHEK cell lines, respectively. 81, 864 sites occurred within a domain present in each of the seven sets of human TADs. One should note that the domains within which 81, 864 sites occur might have different boundaries across two different cell lines. Gene ontology was performed on the genes (5,662) nearest to the 81, 864 sites, revealing associations with development terms and immune system-related terms such as somatic stem cell maintenance, aorta development, Fc receptor signaling, blastocyst development, trophectodermal cell differentiation (Table S3 in Additional file 1).

### “TAD-lncRNAs”: LncRNAs as predictors for topologically associated domains

**Fig. 4:**
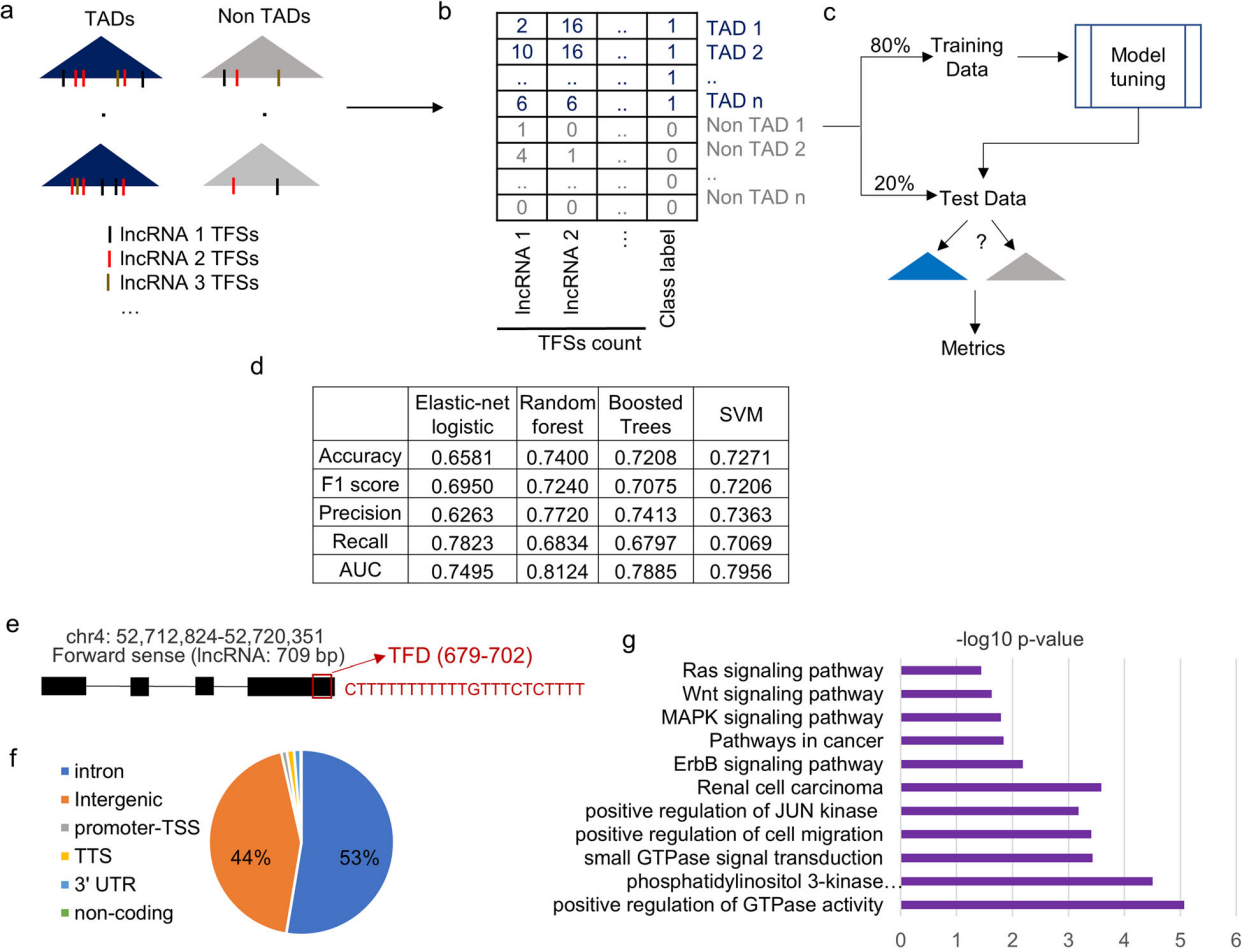
LncRNA:DNA triplex-forming sites as predictors for TADs. (**A**) Triplex-forming sites (TFSs) in *n* TADs and in the background set consisting of *n* randomly selected genomic regions, which do not overlap with TADs. (**B**) The frequency of TFSs for lncRNAs is used as features in a prediction problem, where TADs and the random regions have class labels “1” and “0”, respectively. (**C**) The predictive models are trained on the training set (80 % of 2*n*) to determine the appropriate model parameters. The model performances are computed on the test data (20 % of 2n). (**D**) Prediction accuracies and four other metrics of the predictive models. The values are averaged across the six cell lines (**E**) TAD-lncRNA *DANCR* with its triplex-forming domain (TFD) located from base pair position 679 to 702. (**F**) Genomic annotation of locations of the TFSs of *DANCR* in GM12878 cell line. (**G**) Top gene ontology terms associated with the genes nearest to the TFSs of TAD-lncRNA *DANCR* in the GM12878 cell line. X-axis indicates -log10 p-value

If lncRNA:DNA TFSs are important and enriched features in TADs, they can serve as predictors to differentiate between TADs and other regions. For this, a background set of genomic intervals that were similar in size (a number equal to the number of TADs) was generated by randomly selecting from the genome (excluding the original TAD locations) (Fig. 4A). The TFSs of expressed lncRNAs were also identified in this background set separately for each cell line (Fig. 4A). We used four different feature-based machine learning models to predict the class label of a region of interest (“TAD” or “non-TAD”) by using the frequency of TFSs of expressed lncRNAs in the region as features (Fig. 4B). The models were tuned using a 5-fold cross-validation approach while varying the appropriate model parameters on a training set (80 % of the total pool of data) (Fig. 4C and Table S4 in Additional file 1). Using five different evaluation metrics on the test set, the best performing model was selected (Methods) (Fig. 4D). In this approach, we excluded the H1 cell line because about 83 % of the genomic regions are located within TADs.

On average, the Random Forest model performed the best with an average accuracy of 74 % across the cell lines (Fig. 4D and Table S5 in Additional file 1). The best accuracy achieved were 71.58 %, 71.48 %, 71.20 %, 68.09 %, 70.58 %, and 76.70 % for cell lines GM12878, HeLa, HUVEC, HMEC, NHEK, and IMR90, respectively (Table S5 in Additional file 1 and Fig. 4D). While the best Area Under the Curve (AUC) achieved was 0.81, 0.77, 0.80, 0.68, 0.77, and 0.84 for cell lines GM12878, HeLa, HUVEC, HMEC, NHEK, and IMR90, respectively (Table S5 in Additional file 1 and Fig. 4D). These results show that TFSs of lncRNAs are important and enriched features in TADs and can be used as predictors to discriminate TADs from other regions.

Next, we aimed to identify important “TAD-lncRNAs” which were top predictors in the model performance. To do so, we assigned an “importance score” to each lncRNA based on its discriminating power in the Random Forest model using the “target shuffling” method (Methods). The top 10 “TAD-lncRNAs” for each cell line are shown in Table S6 (Additional file 1). We highlight one particular TAD-lncRNA predictor called *DANCR* or *ENSG00000226950.6* (Fig. 4E) in cell line GM12878. The dominant isoform of *DANCR* with GENCODE id *ENST00000444958.1* is 709 bp long and has a single 23 bp long triplex-forming domain at its 3’ end (Fig. 4E). The triplex-forming domain is rich in T bases and has 2,953 TFSs within TADs (Fig. 4E). Most of the triplex sites of DANCR are either in intergenic (44 %) or intronic regions (53 %) (Fig. 4F). Gene ontology analysis of genes closest to the triplex-forming sites of *DANCR* showed top enrichment in the regulation of GTPase activity (Fig. 4 G) and closely related terms such as JUN kinase activity. Some target genes include Rho GTPase Activating Proteins such as *Arhgap36*, and *Arhgap40*; Fibroblast growth factors such as *Fgf3*, and *Fgf9*. Enrichment in multiple pathways related to cancer such as Ras, Wnt, ErbB, and MAPK (Fig. 4G) was also observed. Some of the important target genes were *Wnt2b, Wnt5b, Wnt5A, Wnt8a* from the *Wnt* pathway, and *Fgf9*, *Mapk1*, *Pak2*, *Igf1*, *Rasa2* from the *Ras* pathway. If TFSs are used as anchors by TAD-lncRNAs to mediate chromatin organization, their deregulation such as *DANCR* can disrupt the formation of TFSs and may alter chromatin organization. Consequentially, it may contribute to diseases including cancer.

## Discussion

Our findings reveal that lncRNA:DNA TFSs are enriched in TADs, their boundaries, and loop anchors. However, TFSs are roughly evenly distributed across TADs indicating no preference for specific regions of TADs. The normalized coverage of TFSs is slightly negatively correlated to the size of domains. Many previously reported TFSs of lncRNAs in vivo such as *Fendrr, Khps1, and PARTICLE* [25–27] are primarily located in promoter regions of genes. In such cases, lncRNAs use TFSs as anchors to transport protein complexes to the specific target regions for direct gene regulation. On the other hand, lncRNA *Firre* mediates chromosomal contacts by interacting with the DNA at non-promoter regions [17]. Interestingly, these interaction sites of *Firre* have high triplex-forming potential [28]. We found that lncRNA:DNA TFSs are not only located in promoter regions, but also positioned in other functional elements such as intergenic, intronic, and 3’UTR regions. In addition to serving as a “dock” located at promoters for lncRNAs to transport protein complexes, our observations suggest a broader role of TFSs. For instance, lncRNA:DNA TFSs located in intergenic and intronic regions may act as anchors to mediate chromosomal contacts in TADs. TFSs located in 3’UTR may be involved in post-transcriptional gene regulation. We also observed the absence of correlation between the TFSs and CTCF sites and it is most likely because CTCF are enriched in boundaries compared to internal regions of TADs, while TFSs showed no preference between boundaries compared to internal TAD regions. Even though this observation doesn’t prove that TFSs have a secondary role in specification and “protection” of the boundaries, we can provide some speculation of a potential link between the specification of the boundaries and the TFSs.

Not all the lncRNAs but about 46 % of the expressed lncRNAs were found to form triplex structures with the DNA. A single lncRNA can form triplex structures with many regions of the DNA via one or two TFDs. The presence of only one or two TFDs that can interact with many regions of the DNA indicates that it is a nonrandom phenomenon. It may be appropriate to compare this observation to the mechanism in which a single transcription factor regulates hundreds of genomic regions by recognizing and binding a consensus short motif located at different regions of the DNA. In the case of lncRNAs, they may use the triplex sites as anchors and mediate 3D organization in various ways such as forming loops, regulating promoter-enhancer interactions, defining chromosome contacts. There was no relationship between the expression patterns of lncRNAs across multiple cell lines and their TFDs and TFSs indicating lncRNA:DNA triplex formation as a general mechanism of action used by lncRNAs across multiple cell lines.

Here, we report the first genome-wide study that demonstrates that lncRNA:DNA TFSs are important features in the context of 3D chromatin organization. LncRNA:DNA TFSs can be used as predictors in machine-learning models to discriminate TADs from other genomic regions. However, it is not certain whether the formation of the triplex sites causes the specification of TADs or the TFSs only plays a role in maintaining the internal organization of TADs. We suspect the two roles to be not mutually exclusive. It is important to note that the discrimination of TADs in a cell line was done using the TFSs of lncRNAs expressed in that cell line only. We used this approach because even if a lncRNA has the potential to form TFSs, it is only relevant if exhibits an expression in the cell line of interest. We also identified top TAD-lncRNAs by scoring the lncRNAs based on the degree of “contribution” of their TFSs to the discriminating power of the machine-learning model. Several lncRNAs with annotated names were identified as important TAD-lncRNAs (Table S6 in Additional file 1). We highlighted one such lncRNA called *DANCR*, which has been shown to have roles in multiple types of cancer. If TFSs are used as anchors by TAD-lncRNAs such as *DANCR* to mediate chromatin organization, their deregulation can disrupt the formation of TFSs and may alter chromatin organization contributing to diseases including cancer.

Our analysis supports the idea that lncRNA:DNA triplex formation is an important mechanism through which lncRNA can exert their function of mediating 3D chromatin organization. Many instances of lncRNAs involved in chromatin regulation via the formation of triple helices with DNA in specific regions have been validated. For example, *HOTAIR* binds to DNA and recruits PRC2 and LSD1-CoREST [29], lncRNA *MEG3* regulates different pathways by the formation of triple helices [30], *Fendrr* recruits PRC2 via RNA:DNA triplex formation [31]. The prediction of RNA:DNA triplex sites is a challenging task, we used triplexator tool to generate the lncRNA:DNA TFSs. We used this approach to be consistent with previous analyses [28, 32] and found it to be in agreement with genome-wide ChIRP-Seq peaks of some lncRNAs. For example, some TFSs of lncRNAs (*TUG1, MEG3, Fendrr, HOTAIR*) with DNA have been experimentally validated using methods such as electrophoretic mobility shift assay were consistent with the prediction by triplexator [28]. Furthermore, significant overlap between sites predicted by triplexator and peaks from ChIRP-Seq of various lncRNAs was found [28]. Convolutional neural networks were used to predict the DNA binding sites (obtained using ChIRP-Seq) of various lncRNAs [32] with good accuracies. It was found that 82 % of the DNA sequence motifs (kernels) learned by the model from lncRNA ChIRP-Seq peaks formed triplex structures (predicted using triplexator) with lncRNAs of interest [32]. The same study experimentally validated new triplex-forming sites of lncRNAs *HOTAIR* and *TUG1* [32].

## Conclusions

In summary, we report that lncRNA:DNA TFSs are enriched at specific locations in relationship to TADs, which are the primary units of chromatin organization. LncRNA:DNA TFSs are enriched in TADs, their boundaries, and loop anchors. However, TFSs are evenly distributed across different regions of a TAD. TFSs and are located not just in promoter regions but also in intergenic, intronic, and 3’UTR regions. This indicates that TFSs may have a bigger role in mediating chromatin organization beyond direct gene regulation via promoter interaction. These TFSs are important and enriched features of TADs since they can be used as predictors to discriminate TADs from other genomic regions. Our observations are consistent with the idea that lncRNA:DNA triplex formation is a general mechanism of action used by some lncRNAs, not just for transportation of protein complexes but to mediate 3D chromatin organization.

## Methods

### TADs, boundaries, and loop locations

Locations of TADs, boundaries of TADs, and loop anchors from 6 different human cell lines (GM12878, HeLa, HMEC, HUVEC, IMR90, and NHEK) were obtained from a study by Rao et al. [1] and that of H1 cell lines was acquired from a study by Dixon et al. [33]. There were no loop anchor locations for the H1 cell line. The boundaries and loop anchors in these studies were identified from HI-C data with kilobase resolution. However, the downloaded locations were one bp long. We extended the length of boundaries and loop anchors on both ends by 10 kb to make each TAD boundary to be 20 kb long.

### LncRNA sequences and their expression

The lncRNA sequences were downloaded from the GENCODE project. The expression profiles of long noncoding RNAs (lncRNAs) were collected from ENCODE project (https://www.encodeproject.org). The expression profiles were indicated as TPM (Transcripts per Kilobase Million). For lncRNAs that have multiple isoforms, we considered the one with the highest number of triplex-forming sites with the hg19 genome. Any lncRNA with TPM (Transcripts per Kilobase Million) value > 5 were considered “expressed”.

### Clustering analysis

To cluster the lncRNAs based on their expression in seven human cell lines, we computed the z-scores of the lncRNAs across the cell lines. HOPACH hierarchical [21] clustering was performed using R hopach package.

### Generation and enrichment analysis of lcnRNA:DNA TFSs

The triplex-forming sites (TFSs) of lncRNAs were determined by aligning the lncRNA sequences to the hg19 genome by triplexator tool setting the minimum triplex feature-length to 20. For lncRNAs with multiple isoforms, we used the isoform with the maximum number of TFSs. To assess whether the triplex-forming sites of lncRNAs are enriched in regions of interest (TADs or boundaries or anchors), we computed the observed coverage (or number of base pair overlaps) of regions of interest with the triplex-forming sites using bedtools [34].

An expected coverage was generated by randomly permuting the real triplex-forming sites (TFSs) within the genome and computing the coverage of this random set with the regions of interest. This random shuffling was performed 1000 times, for each shuffled set; an expected coverage was obtained to generate a distribution of expected coverage. These distributions were tested for a normal distribution for all the seven cell lines using the Anderson-Darling normality test. P-values were computed using the observed coverage in the region of interest and estimated parameters of the normal distribution. To compare differential positional preference of the TFSs of lncRNAs between the TADs boundaries and regions within the TADs, the positions of TFSs that overlap with boundaries of TADs was randomly permutated so that they fell only within the TADs (but excluding the boundary regions).

### Functional elements annotation

The functional annotation of TFSs was done using the “annotatePeak.pl” module in HOMER tool [35]. According to HOMER, the NCBI RefSeq transcript definitions from the UCSC genome browser are used to derive the functional annotations of different genomic regions. HOMER uses promoters from all transcripts of a gene. It defines promoters as genomic intervals that encompass 1 kb upstream and 100 bp downstream of TSSs. To compare the enrichment of TFSs in different functional genome regions, we computed the density of TFSs in each type of functional element as the number of TFSs in every 10 kb region of that particular functional element. This was done simply done by multiplying the ratio of overlapping TFSs and summed length of the functional element by 1000. This normalization was done to make appropriate comparisons between different types of functional elements which differ vastly in size. Overlapping genomic regions annotated to the same functional element were merged before computing the summed length.

### Training machine learning models

For a cell line, we considered all the *m* lncRNAs (lncRNA_1_, lncRNA_2_, …, lncRNA_m_) which had at least one triplex-forming site in at least one of *n* TADs (TAD_1_, TAD_2_,., TAD_n_). We generated *n* random genomic intervals (non-TAD_1_, non-TAD_2_, ., non-TAD_n_) which did not overlap with any of the TADs. TFSs of the same *m* lncRNAs were determined on these *n* random genomic intervals. We posed a supervised machine-learning problem, where the class labels of *2n* samples (*n* TADs of the class label “1” and *n* non-TADs with the class label “0”) can be predicted using the count of TFSs of the *m* lncRNAs as features. Four different models (Table S5 in Additional file 1) were trained to determine the best model parameters using a training set (80 % of *2n* samples) with 5-fold cross-validation. The performances of the models were reported by predicting on the test samples (20 % of *2n* samples). The parameters tuned in the models are given in Table S4 in Additional file 1. All models were implemented using the R language, CARET [36], and GLMET [37] packages.

In the context of determining “TAD-lncRNAs”, we determined the “importance” of a lncRNA (lncRNA_i_) in the machine learning model. To do so, only the counts of TFSs of lncRNA_i_ were randomly shuffled across the samples in the original test set. We computed the accuracy on the shuffled test set using the best Random Forest model. The difference between the accuracies on the original test set and shuffled test set was used as the importance of the lncRNA_i_. The higher the difference, the higher the importance score of the lncRNA.

## Supplementary information

**Additional file 1**

## Data Availability

The datasets used and/or analyzed during the current study are available from github page https://github.com/lncRNAAddict/Triplex.
