# Supplementary material for: LncRNA:DNA triplex-forming sites are positioned at specific areas of genome organization and are predictors for Topologically Associated Domains
Source: BMC Genomics. 2021 May 28;22:397. doi: 10.1186/s12864-021-07727-7 (PMC8164242; doi:10.1186/s12864-021-07727-7)
Supplement: Supplementary file 1 — Additional file 1 [file 12864_2021_7727_MOESM1_ESM.pdf]

Table S1: Proportion of the genome covered by TADs, TAD boundaries, and loop anchors

	Proportion of genome covered (%)		
**Cell line/**	**TADs**	**Boundaries**	**Loop Anchors**
**H1ESC**	83.18	0.95	
**GM12878**	53.39	2.89	3.15
**HeLa**	47.53	2.22	3.56
**HMEC**	40.07	2.27	2.28
**HUVEC**	49.19	2.16	4.17
**IMR90**	65.75	2.43	3.19
**NHEK**	55.98	3.08	5.9

Table S2: P-values from Anderson-Darling Normality test for the estimated coverage of TFSs. An expected coverage was generated by randomly permuting the real triplex forming sites (TFSs) within the genome (for Boundary vs Inside the TFSs were shuffled within the internal regions of TADs). All p-values are > 0.01 indicating that all estimated distributions follow a Normal distribution.

Cell Line	Domain vs genome	Boundary vs genome	Loop Anchor vs genome	Boundary vs Inside
GM12878	0.024	0.8201	0.5478	0.427
HELA	0.171	0.04859	0.6233	0.470
HUVEC	0.7937	0.3247	0.6173	0.3247
NHEK	0.751	0.8849	0.1414	0.1591
H1ESC	0.6616	0.736	NA	0.2578
HMEC	0.1762	0.3937	0.898	0.7135
IMR90	0.663	0.2397	0.2967	0.2397

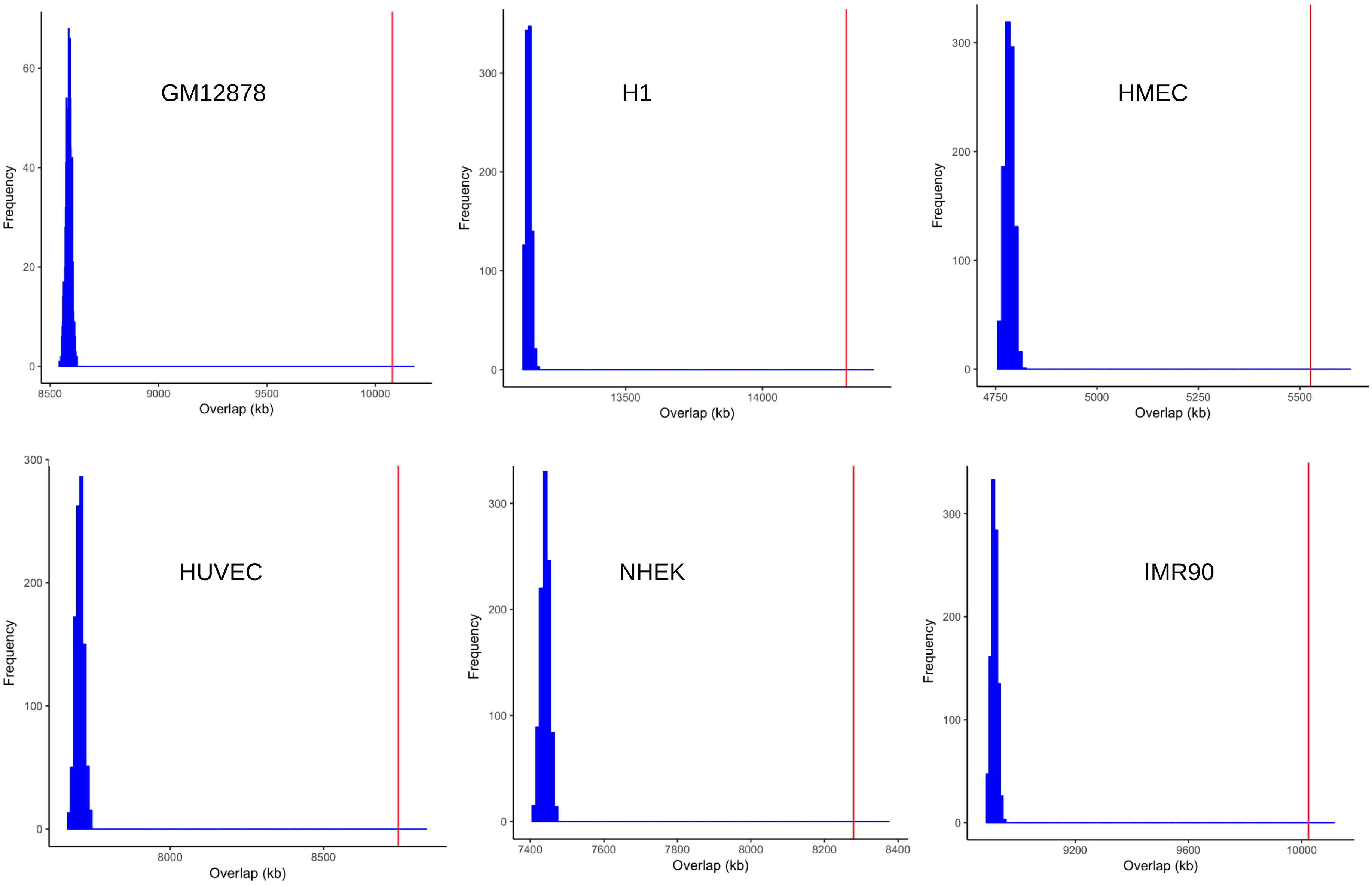

Fig S1.Triplex forming sites (TFSs) are enriched in domains. Distribution of expected coverage (blue) versus the observed coverage (vertical red line) of TFSs in domains are shown for six cell lines. All p-values were less than 0.001.

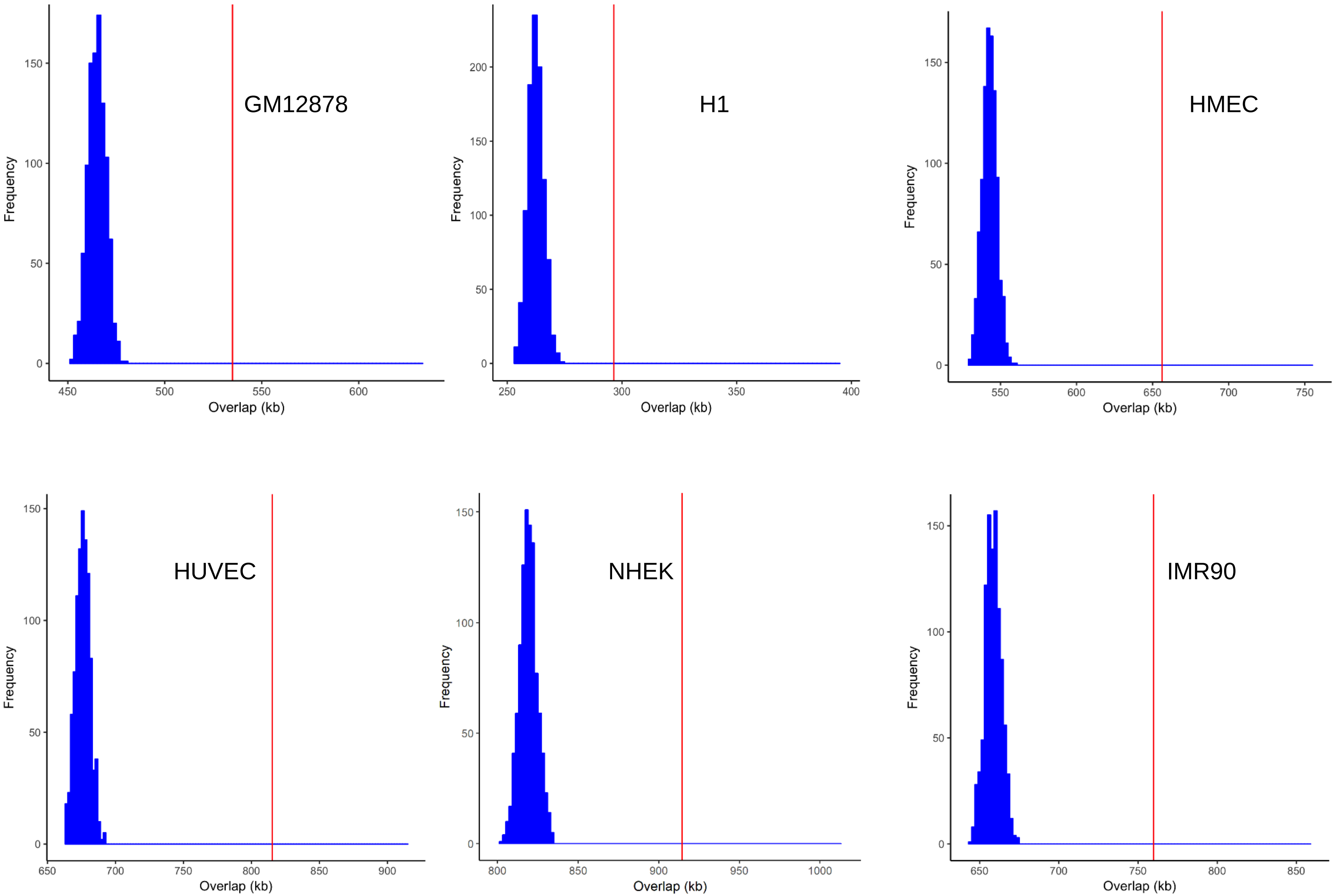

Fig S2.Triplex forming sites (TFSs) are enriched in boundaries of TADs. Distribution of expected coverage (blue) versus the observed coverage (vertical red line) of TFSs in boundaries of TADs are shown for six cell lines. All p-values were less than 0.001.

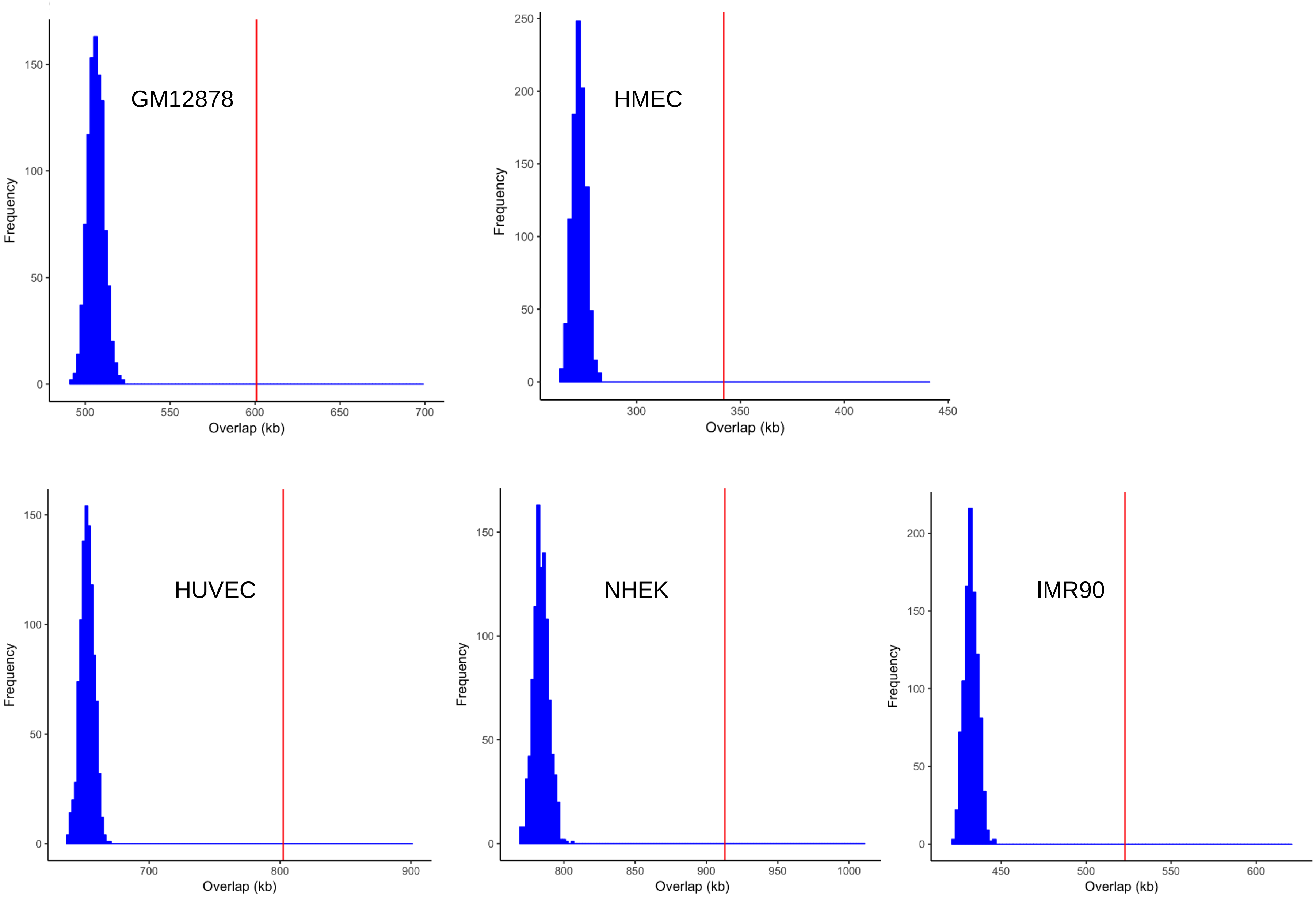

Fig S3.Triplex forming sites (TFSs) are enriched in loop anchors of TADs. Distribution of expected coverage (blue) versus the observed coverage (vertical red line) of TFSs in loop anchors of TADs are shown for five cell lines. All p-values were less than 0.001.

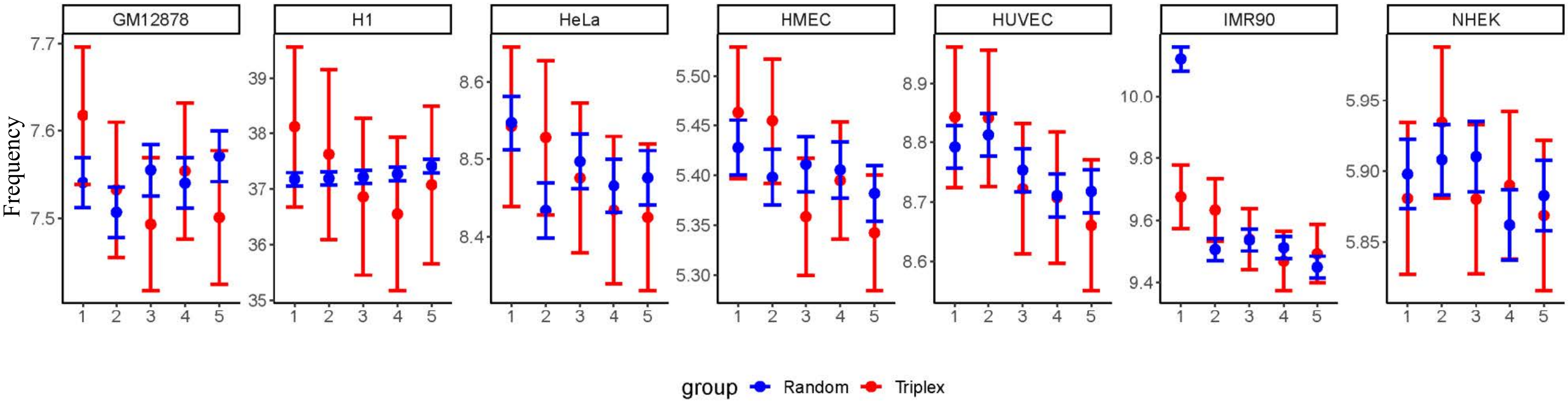

Fig S4.Triplex forming sites (TFSs) are evenly distributed across a TAD. Expected frequency of TFSs (red) versus the observed frequency (red) of TFSs in five different bins (1, 2, 3, 4, 5) along the TADs. Bin 1 is closest to the boundary, bin 5 is the middle portion of TAD. Frequencies of observed TFSs is evenly distributed across TADs and not significantly different from expected frequencies (p-value > 0.1 using Kolmogorov-Smirnov test).

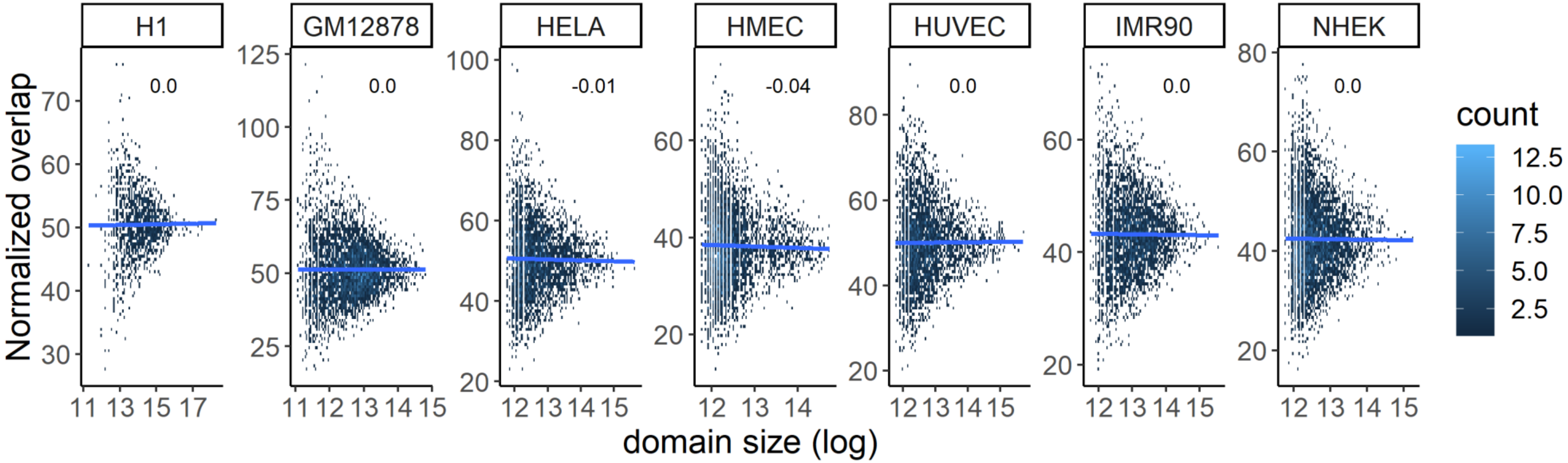

Fig S5. Expected coverage vs size of TADs. TFSs were randomly positioned across the genome. Plot displays scatter plot of TADs sizes (x-axis) and normalized overlap by the randomized sites (coverage by TFSs/TADs' size). The Pearson correlation coefficients are indicated for each cell line.

Table S3. Gene ontology terms associated with genes closest to the triplex-forming sites located in TADs that are shared in many cell types.

# Term Name	Binom Rank	Binom Raw P-Value	Binom FDR Q-Val
Fc receptor mediated stimulatory signaling pathway	127	4.11E-120	3.38E-118
Fc-gamma receptor signaling pathway involved in phagocytosis	130	1.61E-119	1.29E-117
Fc-gamma receptor signaling pathway	131	3.27E-119	2.61E-117
phagocytosis	132	8.06E-119	6.37E-117
somatic stem cell maintenance	157	3.66E-105	2.44E-103
keratan sulfate biosynthetic process	185	3.32E-94	1.88E-92
keratan sulfate metabolic process	194	1.75E-90	9.43E-89
phosphate ion transport	199	3.13E-88	1.64E-86
blastocyst development	205	6.28E-86	3.20E-84
aorta development	297	3.89E-66	1.37E-64
regulation of blood coagulation	299	5.56E-66	1.94E-64
apical junction assembly	319	2.97E-63	9.71E-62
aorta morphogenesis	326	6.88E-62	2.20E-60
regulation of cell fate specification	423	1.82E-48	4.49E-47
JAK-STAT cascade	448	1.82E-45	4.25E-44
trophectodermal cell differentiation	451	2.43E-45	5.63E-44
nucleobase-containing small molecule interconversion	528	1.24E-39	2.45E-38
regulation of transcription from RNA polymerase II promoter in response to hypoxia	561	6.72E-38	1.25E-36
negative regulation of insulin receptor signaling pathway	602	2.25E-35	3.91E-34
cellular response to interleukin-4	648	1.34E-33	2.15E-32
parasympathetic nervous system development	665	9.42E-33	1.48E-31
dorsal/ventral axis specification	693	8.09E-32	1.22E-30
lateral ventricle development	795	1.28E-28	1.68E-27
response to lipoprotein particle stimulus	885	2.94E-26	3.47E-25
regulation of cholesterol storage	904	1.00E-25	1.16E-24
regulation of extrinsic apoptotic signaling pathway via death domain receptors	1007	1.47E-23	1.52E-22
leukocyte aggregation	1078	2.37E-22	2.30E-21
positive regulation of blood coagulation	1290	5.36E-19	4.34E-18
positive regulation of coagulation	1350	3.74E-18	2.89E-17
positive regulation of T-helper cell differentiation	1415	2.71E-17	2.00E-16
chronic inflammatory response	1890	2.05E-12	1.13E-11
regulation of plasminogen activation	2001	1.66E-11	8.64E-11

Table S4. Model parameters that are tuned. First column is the model's name, second column indicates model parameters. All possible options of the parameter that were tested are provided in parenthesis.

Model	Parameters
Elastic-net logistic regression	α (0.05, 0.5, 0.1), λ (default sequence of values chosen by glmnet package)
Random Forest	mtry(0, n/40, 2*n/40, ….n), number of trees (200)<br>where n = number of features
Boosted Trees	trials(1, 2, 4, …100), winnow (TRUE, FALSE)
Support Vector Machines (SVM)	kernel(radial basis), σ(three values as suggested by sigest function in kernlab R package), C(2^{-4}, 2^{-3}, ….,2^{8})

Table S5. Performances of four models in seven cell lines.

GM12878

	Logistic Regression	Random Forest	Boosted Tree	SVM radial basis kernel
**Accuracy**	67.33%	72.57%	70.64%	71.03%
**F1 Score**	0.7101	0.7209	0.7044	0.7104
**Precision**	0.6382	0.7337	0.7092	0.71
**Recall**	0.8002	0.7086	0.6998	0.7108
**AUC**	0.7592	0.8111	0.7931	0.7919

H1

	Logistic Regression	Random Forest	Boosted Tree	SVM radial basis kernel
**Accuracy**	69.62%	90.19%	90.58%	86.54%
**F1 Score**	0.7492	0.8957	0.9014	0.8638
**Precision**	0.6378	0.9563	0.9451	0.874
**Recall**	0.9077	0.8423	0.8615	0.8538
**AUC**	0.853	0.9538	0.9544	0.9289

HMEC

**Accuracy**	62.83%	66.69%	67.18%	68.09%
**F1 Score**	0.6667	0.6453	0.6471	0.6734
**Precision**	0.6043	0.6889	0.6998	0.6897
**Recall**	0.7433	0.6087	0.6017	0.6578
**AUC**	0.7017	0.7381	0.7368	0.7363

HUVEC

**Accuracy**	63.44%	71.12%	66.77%	69.94%
**F1 Score**	0.6741	0.6914	0.6647	0.6905
**Precision**	0.6081	0.7424	0.6707	0.7116
**Recall**	0.7563	0.647	0.6588	0.6706
**AUC**	0.72	0.7959	0.7475	0.7612

NHEK

**Accuracy**	62.72%	70.58%	66.18%	68.98%
**F1 Score**	0.6508	0.6651	0.6116	0.6599
**Precision**	0.612	0.7719	0.7183	0.7302
**Recall**	0.6949	0.5843	0.5326	0.6019
**AUC**	0.699	0.7718	0.7274	0.7522

HELA

**Accuracy**	65.09%	70.98%	68.39%	69.32%
**F1 Score**	0.6889	0.7003	0.6755	0.6917
**Precision**	0.6212	0.7239	0.6939	0.6952
**Recall**	0.773	0.6782	0.658	0.6882
**AUC**	0.7257	0.7739	0.7511	0.7672

IMR90

**Accuracy**	69.4200%	75.2600%	73.8800%	75.0700%
**F1 Score**	0.7236	0.7412	0.7300	0.7542
**Precision**	0.6602	0.7770	0.7556	0.7436
**Recall**	0.8005	0.7087	0.7060	0.7651
**AUC**	0.7880	0.8419	0.8091	0.8318

Table S6. Top TAD-lncRNAs. First column is the model's name, second column indicates model parameters. All possible options of the parameter that were tested are provided in parenthesis.

GM12878	Hela	HMEC	HUVEC	IMR90	NHEK
AC096741.2	*DARS-AS1*	*SNHG18*	*AC096741.2*	*AC096741.2*	*CYTOR*
ENTPD1-AS1	*AC011753.2*	*PDXDC2P-NPIPB14P*	*LINC00472*	*AL355075.4*	*AC007406.5*
AC012313.1	*NEAT1*	*AL035071.1*	*C1orf132*	*LRRC75A-AS1*	*AC090673.1*
AC012645.2	*NUTM2A-AS1*	*SNHG12*	*MALAT1*	*SNHG16*	*AC096741.2*
PDXDC2P-NPIPB14P	*AC096741.2*	*LINC01128*	*AC125807.2*	*PDXDC2P-NPIPB14P*	*C1RL-AS1*
LINC01585	*AL359076.1*	*AC087473.1*	*AL445248.1*	*AC090673.1*	*TMEM147-AS1*
TRAF3IP2-AS1	*NNT-AS1*	*AC068768.1*	*LINC01013*	*SNHG12*	*NUTM2A-AS1*
DANCR	*LINC01355*	*MALAT1*	*THUMPD3-AS1*	*NEXN-AS1*	*SNHG16*
SNHG10	*AL357992.1*	*MHENCR*	*LINC01389*	*TUG1*	*AC027288.3*
CD27-AS1	*MIR17HG*	*AC092611.2*	*AC022784.7*	*NUTM2A-AS1*	*AC016747.1*
